# Effects of Static Heat and Dynamic Current on Al/Zn∙Cu/Sn Solder/Ag Interfaces of Sn Photovoltaic Al-Ribbon Modules

**DOI:** 10.3390/ma11091642

**Published:** 2018-09-07

**Authors:** Kuan-Jen Chen, Fei-Yi Hung, Truan-Sheng Lui, Wen-Yu Lin

**Affiliations:** 1 The Instrument Center, National Cheng Kung University, Tainan 701, Taiwan; kjchen1982@gmail.com; 2 Department of Materials Science and Engineering, National Cheng Kung University, Tainan 701, Taiwan; luits@mail.ncku.edu.tw (T.-S.L.); frankielin0216@gmail.com (W.-Y.L.)

**Keywords:** Al ribbon, electroplated, electromigration, intermetallic compound

## Abstract

This present study applied Cu∙Zn/Al ribbon in place of a traditional Cu ribbon to a photovoltaic (PV) ribbon. A hot-dipped and an electroplated Sn PV ribbon reflowed onto an Ag electrode on a Si solar cell and estimated the feasibility of the tested module (Ag/Solder/Cu∙Zn/Al). After bias-aging, a bias-induced thermal diffusion and an electromigration promoted the growth of intermetallic compounds (IMCs) (Cu_6_Sn_5_, Ag_3_Sn). To simulate a photo-generated current in the series connection of solar cells, an electron with Ag-direction (electron flows from Ag to Al) and Al-direction (electron flows from Al to Ag) was passed through the Al/Zn∙Cu/Solder/Ag structure to clarify the growth mechanism of IMCs. An increase in resistance of the Ag-direction-biased module was higher than that of the Al-direction biased one due to the intense growth of Cu_6_Sn_5_ and Ag_3_Sn IMCs. The coated solder of the electroplated PV ribbon was less than that of the hot-dipped one, and thus decreased the growth reaction of IMCs and the cost of PV ribbon.

## 1. Introduction

Photovoltaic (PV) ribbon structure is made of a Cu ribbon coated with an alloy solder. In order to promote solar PV systems, using lead-free and low-cost solder in the field of the PV ribbon are important topics [1,2,3].

Low-cost Sn–Cu alloys are served as a candidate solder to substitute for Sn–Pb solder due to their good weldability with Cu [4,5]. However, Cu_6_Sn_5_ intermetallic compound (IMC) at the Sn-xCu solder/Cu interface grows rapidly under current-transport and bias-induced Joule heat, deteriorating the conductivity of an electronic device [6,7]. IMC is not formed in the Sn–Zn alloy structure, which has good conductivity [8]. A previous study reported that adding more Zn to the Sn-xZn solder could reduce the resistivity and the cost of the solder [9]. However, highly active Zn is easily oxidized to form scum in the molten Sn–Zn alloy, decreasing solder weldability. Using a protective atmosphere or a flux during soldering, or adding appropriate alloying elements (Al, Cr, In) to a solder can improve the weldability between the solder and a Cu ribbon [10,11].

During the hot-dip process, the molten solder/Cu ribbon interface is a solid-liquid reaction system [12]. With the melting and diffusion of solid Cu atoms, the Cu_6_Sn_5_ compound is formed at the interface between the solder and the Cu ribbon. The Cu_6_Sn_5_ IMC grows rapidly after biasing for a long duration, which deteriorates the conductivity of the PV module. Therefore, the Cu ribbon in PV ribbon manufacturing has room for improvement.

Al metal can be used for replacing the Cu ribbon due to its good conductivity, light weight and low cost. There are few studies on the interfacial reaction between the solder alloy and Al substrate in PV module application [13]. The Al ribbon does not easily react with other metals to form IMC for bonding, and, thus, using a Zn-containing solder alloy is necessary for solder/Al connection. The liquid Zn will penetrate into the high-angle grain boundaries of the Al ribbon, indicating that this bonding behavior does not generate IMC. Note that Zn content in the solder needs to exceed 50 wt.%, which will have significant penetrating behavior. However, a molten solder with a higher Zn content was easier to oxide to form scum, resulting in the waste of molten solder. In the present study, an electroless plating Zn Al-ribbon is used for PV ribbon fabrication, and investigates the series resistance and interfacial microstructure of PV module structure (Al/Zn∙Cu/Solder/Ag).

## 2. Experimental Procedure

For the metal matrix in the PV ribbon, the 1050 Al-ribbon was used to substitute for the traditional Cu-ribbon (Table 1). The Zn and Cu metals were sequentially coated onto the Al-ribbon by electroless plating and electroplating, respectively, so that the Al-ribbon has a Cu surface (Cu∙Zn/Al). Then, the Sn layer was coated onto the Cu∙Zn/Al ribbon by electroplating (EP) and the hot-dipping (HD) method to acquire the Sn PV Al-ribbon (Sn/Cu∙Zn/Al). The EP (265 °C for 20 s) and the HD (320 °C for 15 s) PV ribbons were reflowed onto an Ag electrode on a Si solar cell. The detailed procedure described in our previous report [2], the peeling force test was executed to estimate the bonding reliability of applying Sn/Cu∙Zn/Al PV ribbons for Si solar cells. The biasing test was performed under a fusing current of 70% (12.6 A) for 72 h to investigate the series resistance, the interfacial diffusion and electrical migration behaviors of PV modules. The interfacial microstructures and element composition ratio were examined using an ultra-high-resolution field-emission scanning electron microscope (UHRFE-SEM, ZEISS, Jena, Germany) and an energy-dispersive X-ray spectrometer (EDS, ZEISS, Jena, Germany). An electron probe microanalyzer (EPMA, JEOL, Akishima, Japan) was used to analyze the interfacial diffusion behavior of the PV modules. The series resistances of the PV module structure (Ag/Sn/Cu∙Zn/Al) were calculated according to Ohm’s law.

## 3. Results and Discussion

### 3.1. Connection Characteristics of PV Al-Ribbon Modules

**Figure 1:**
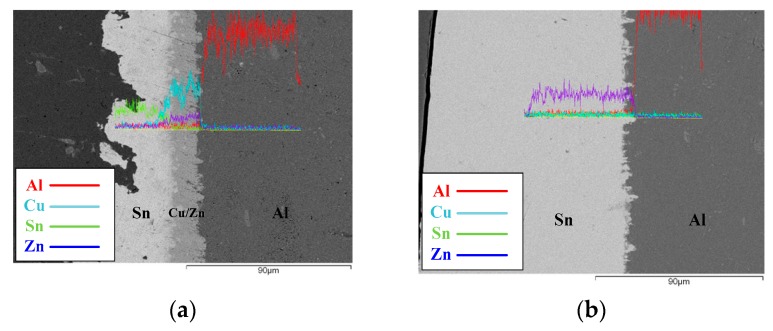
Interfacial images of (**a**) the electroplating (EP) and (**b**) the hot-dipping (HD) photovoltaic (PV) Al-ribbons.

**Figure 2:**
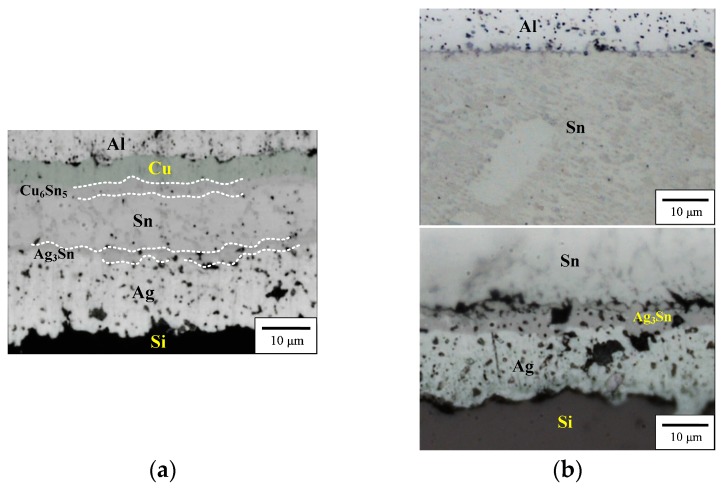
Interfacial images of (**a**) the EP and (**b**) the HD PV modules.

Figure 1 shows the interfacial images of the EP and the HD PV Al-ribbon (Sn/Cu∙Zn/Al). The thickness of the electroplated Sn layer is thinner than that of the hot-dipped Sn layer. Although the Zn layer in both the EP and HD PV Al-ribbon is not observed at the Cu/Al interface, it was detected in the Zn signal from the EDS analysis (Figure 1a). Note that the Cu layer does not exist in the HD PV Al-ribbon (Figure 1b), which is attributed to Cu metal being remitted into the molten Sn solder during the hot-dipping process. The Cu_6_Sn_5_ IMC grows at the Cu/Sn interface and the Ag_3_Sn IMC combines the PV Al-ribbon and Si solar substrate (Figure 2a). Compared with the EP PV module, the HD PV module has a thicker Ag_3_Sn IMC at the Sn/Ag interface, which is associated with a higher reflow temperature (Figure 2b).

**Figure 3:**
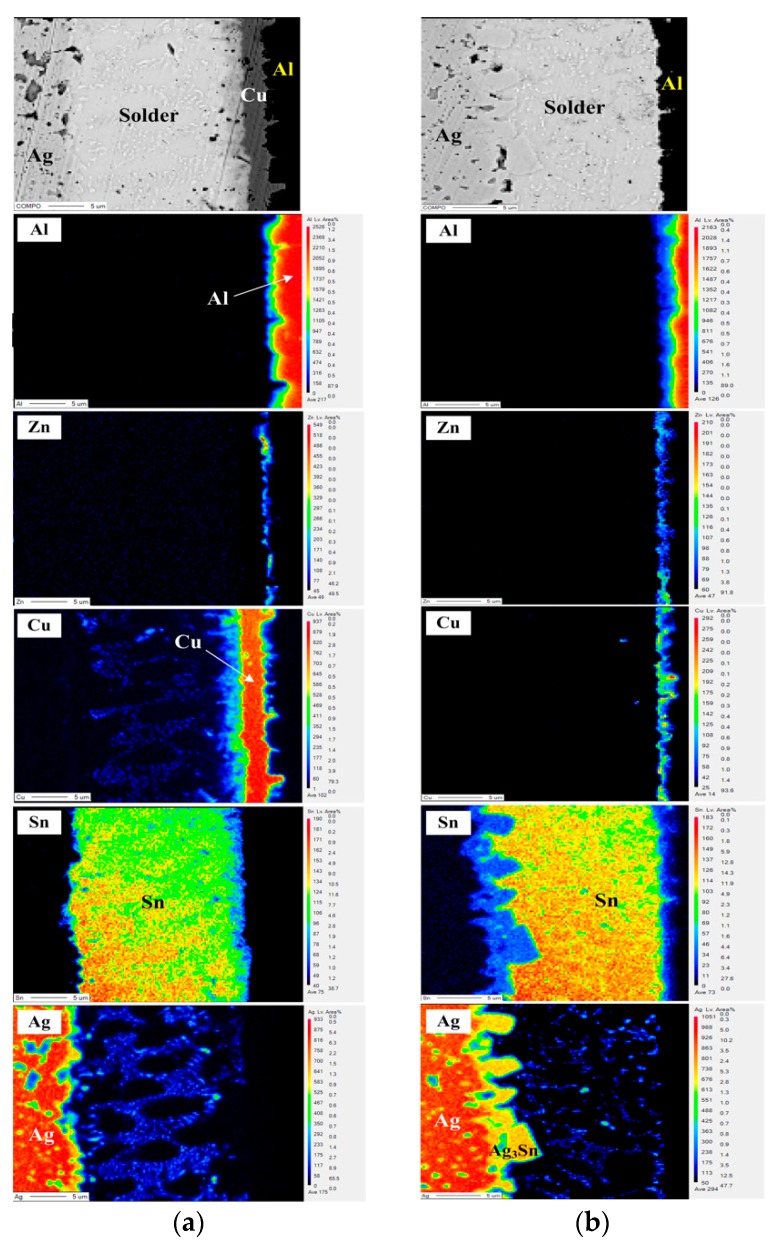
Electron probe microanalyzer (EMPA) images of the interfacial regions for (**a**) the EP and (**b**) the HD PV modules.

To determine the elemental (Al, Zn, Cu, Sn and Ag) distribution in the PV modules, EPMA was used to examine the interfacial structures (Figure 3). The electroless plated Zn layer is detected at the Cu/Al interface that can help to form an Al-ribbon with a Cu metal surface. As shown in Figure 3a, Ag atoms combined with Sn to form Ag_3_Sn IMC at the Ag/Sn interface, and also diffused into the electroplated-Sn layer to form the eutectic structure (β-Sn + Ag_3_Sn) (Ag signal diagram). The Cu_6_Sn_5_ IMC forms at Sn/Cu interface and a trace amount of Cu atoms diffused into the Sn layer along the grain boundaries of Ag_3_Sn (Cu signal diagram). Notably, the signal intensity of Cu is lower than that of Ag, because the Cu_6_Sn_5_ IMC layer can restrain Cu atoms diffusing into the Sn layer. In addition, the diffusion coefficient of Ag (241 cm^2^∙s) was higher than that of Cu (147 cm^2^∙s) and that also affects the contents of Ag and Cu diffused into the solder [14]. In Figure 3b, no obvious signal of the Cu layer is detected in the HD PV module structure, which is attributed to the Cu signal being covered by the Sn signal (Cu signal diagram). Note that the thickness of the Ag_3_Sn IMC layer in the HD PV module (6.86 µm) is thicker than the EP one (3.42 µm), which is attributed to the former having more solder. In addition, the series resistance of the EP and the HD PV modules was measured (Table 2). The series resistance of both of the PV modules was not obviously different. The series resistance does not substantially increase in the manufacturing process from the Al-ribbon to the Sn/Cu∙Zn/Al PV ribbon. The conductivity of the Al-ribbon itself dominates the series resistance of the PV module, indicating that the coated layer (Zn, Cu and Sn) has no significant influence on the conductivity of the PV module.

### 3.2. Electro-Thermal Effect on IMC Evolution of PV Modules

**Figure 4:**
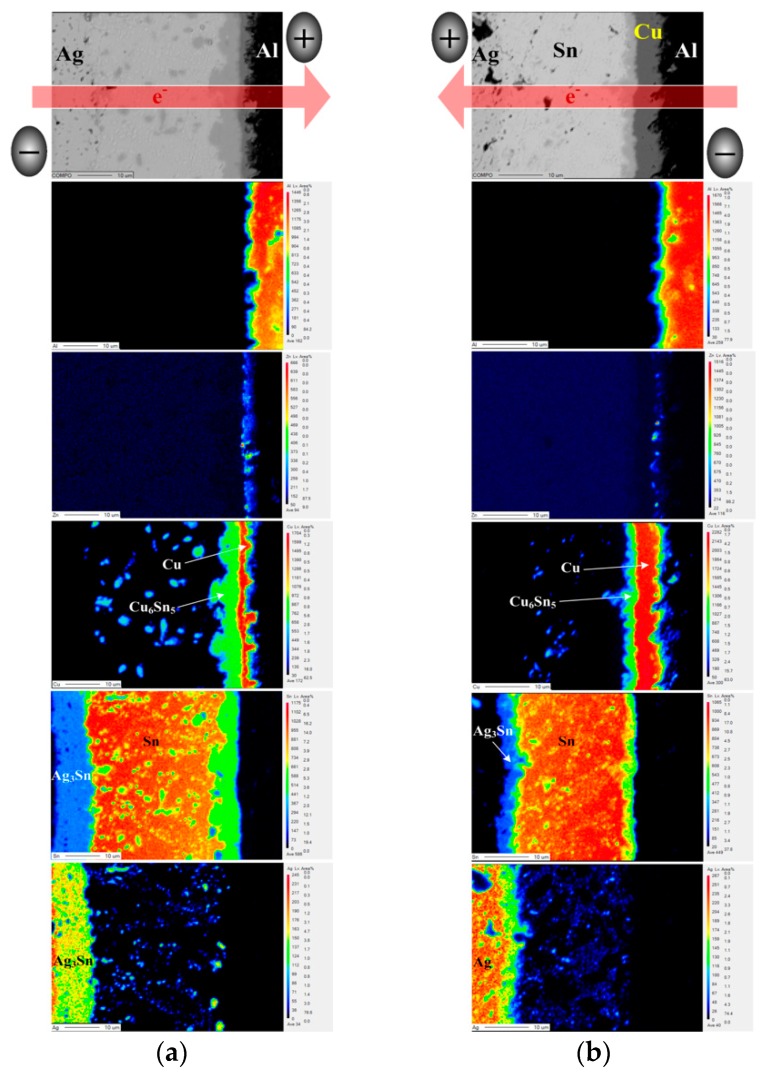
EMPA images of the EP PV module after biasing for 72 h: (**a**) Ag-direction, (**b**) Al-direction.

**Figure 5:**
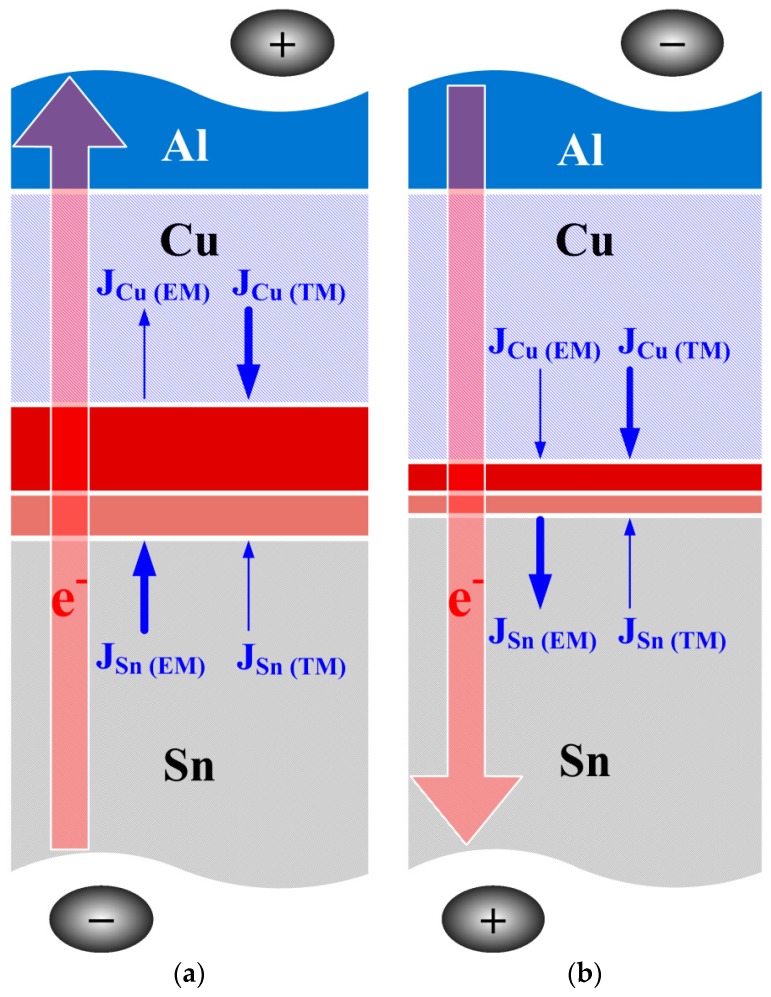
Growth mechanism of Cu_6_Sn_5_ intermetallic compound (IMC) in the EP PV module at the Cu/Sn interface after (**a**) Ag-direction and (**b**) Al-direction biasing.

The bias aging test was performed to estimate the effects of photo-generated current on the series resistance of PV modules. The series resistance of all PV modules increased after biasing for 72 h (Table 3). This result is attributed to a bias-induced Joule heat and an electromigration promoting the growth of Cu_6_Sn_5_ and Ag_3_Sn IMCs at the interface in the module structure. The elemental distribution of interfacial structures in the EP PV module with a bias duration of 72 h is shown in Figure 4. According to the electron flow path, the electron flow from the Al-ribbon to the Ag electrode is defined as “Al-direction”; the opposite is defined as “Ag-direction”. After Ag-direction bias aging (Figure 4a), the Cu_6_Sn_5_ and Ag_3_Sn IMC layers significantly increased compared to that of the unbiased EP PV module (Figure 3a). The electron flow from the Ag electrode to the Al ribbon resulted in an accumulation of Cu_6_Sn_5_ near the positive electrode. A direct current is passed through the PV module structure, which drives the atoms by electro-transport and thermo-transport effects [15]. The flux equation for the given module structure can be written

(1) $$J_{i}=\frac{-D_{i}N_{i}}{RT}\left(\frac{RT\partial \ln N_{i}}{\partial x}+FZ_{i}^{*}E\right)$$

where *D_i_* is the isothermal diffusion coefficient, *N_i_* is the mole fraction of the atom, *F* is Faraday’s content, $Z_{i}^{*}$ is the effective charge and *E* is the electrical field. For the Cu atom, $\frac{RT\partial \ln N_{i}}{\partial x}$ is the chemical potential, which is the main driving force for the growth of Cu_6_Sn_5_. The electromigration force ($FZ_{i}^{*}E$) can be ignored in the Sn–Cu system. However, the flux of Sn electromigration (J_Sn (EM)_) is the main driving force that affected the diffusion of the Sn atom. The flux of Cu thermomigration (J_Cu (TM)_) is not in the same direction as that of the J_Sn (EM)_ (Figure 5a). Therefore, a thermomigration and an electromigration force push Cu and Sn atoms, causing the Cu_6_Sn_5_ accumulation to increase. A large number of Sn atoms reacted with Cu atoms, so the Ag atoms were carried to fill the position where Sn was consumed, resulting in an increment of the Ag_3_Sn IMC layer. In addition, the existing Ag and Cu in the Sn solder matrix will precipitate to form a large number of eutectic structures (β-Sn + Ag_3_Sn + Cu_6_Sn_5_), which deteriorated the module conductivity. As shown in Figure 4b, the kinetic energy of IMC growth in the Al-direction is smaller than that in the Ag-direction. The driving forces of J_Cu (TM)_ and J_Sn (EM)_ are in the same direction, decreasing the kinetic energy of the Cu_6_Sn_5_ growth (Figure 5b). The J_Sn (EM)_ reduced the amount of Sn atoms reacting with Cu atoms to form the IMC. Less Ag atoms diffused toward the negative electrode, reducing the growth behavior of the Ag_3_Sn IMC. Compared with Al-direction biasing, the Ag-direction biasing led to more IMC growth, and, thus, the Ag-direction biased PV module having a higher series resistance.

**Figure 6:**
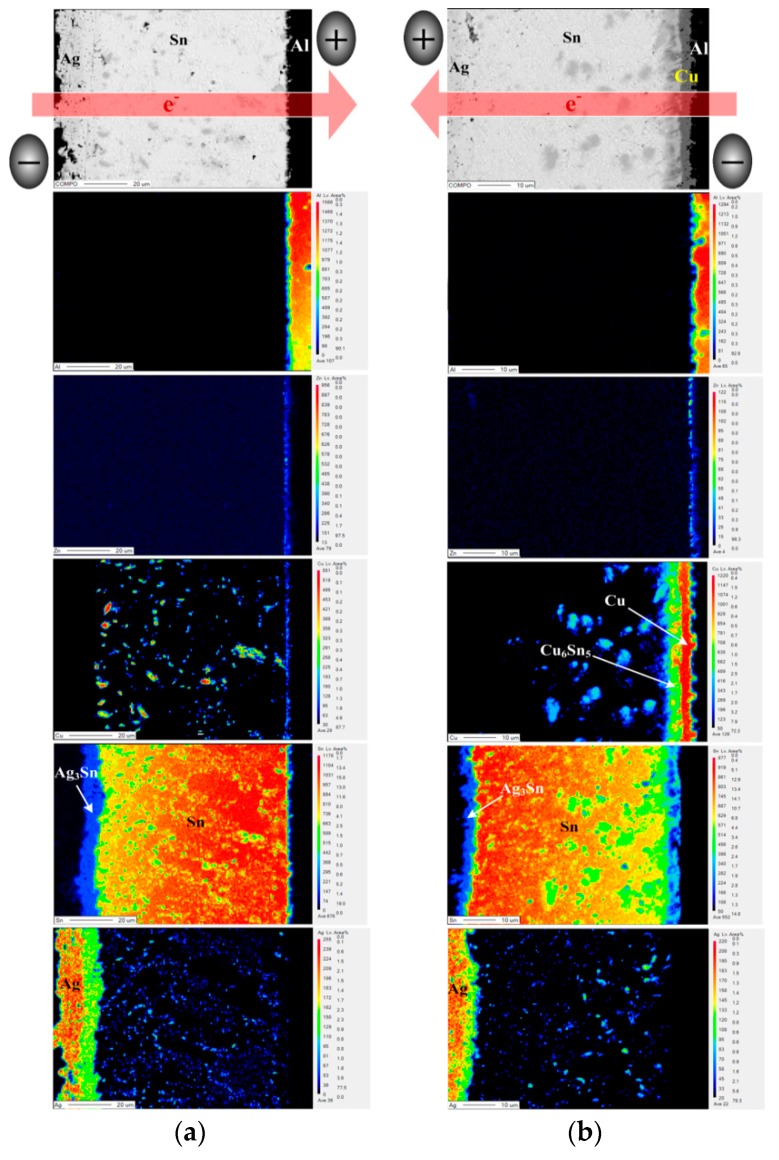
EMPA images of the HD PV module after bias aging: (**a**) Ag-direction and (**b**) Al-direction.

**Figure 7:**
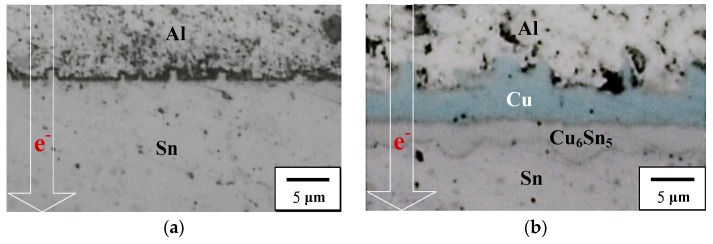
Interfacial microstructures of the HD PV module after Al-direction biasing for (**a**) 24 h and (**b**) 48 h.

Figure 6 shows EPMA images of the interfacial regions for the biased HD PV module. The thickness of the Ag_3_Sn layer in the Ag-direction biased PV module was higher than that in the Al-direction biased one. This result is attributed to current-transport driving Ag and Sn atoms toward the positive electrode, resulting in Ag atoms more easily reacting with Sn to form the Ag_3_Sn IMC. Under an Ag-direction biasing condition (Figure 6a), the Cu metal layer still did not appear in the module structure. During the hot-dip process, the liquid Sn atoms contacted the Cu atoms, which originally bonded on the Cu lattice, reducing the bond strength between the Cu atoms and the other Cu atoms in the crystal to form a metastable Cu–Sn diffusion region. After that, a sufficient amount of the Cu atoms accumulated in the molten Cu–Sn diffusion region, and thus crystallized out the Cu_6_Sn_5_ compound. The Cu_6_Sn_5_ IMC was distributed in the solder matrix, and accumulated toward the negative electrode due to the driving force of J_Cu (TM)_ (Figure 3). Note that the Cu and the Cu_6_Sn_5_ layer appear between the Al ribbon and the Sn solder after Al-direction biasing (Figure 6b). Both Cu and Sn did not react with the Zn layer to form compounds. The J_Cu (EM)_ and J_Cu (TM)_ drove Cu atoms accumulating to form a Cu layer, and then Cu atoms reacted with Sn to form the Cu_6_Sn_5_ layer at the Cu/Sn interface. In addition, the HD PV module was also biased for 24 h and 48 h to clarify the growth behavior of the Cu layer and Cu_6_Sn_5_ IMC (Figure 7). The Cu and Cu_6_Sn_5_ layer did not exist in the module structure after biasing for 24 h (Figure 7a), but appeared after biasing for 48 h (Figure 7b). This indicates that it took over 24 h of the Cu atoms accumulating and diffusing to form the Cu and Cu_6_Sn_5_ layer. The driving force for J_Cu (TM)_ and J_Sn (EM)_ was the same direction, reducing the growth kinetic energy of Cu_6_Sn_5_, and thus Cu_6_Sn_5_ IMC could not over-grow.

**Figure 8:**
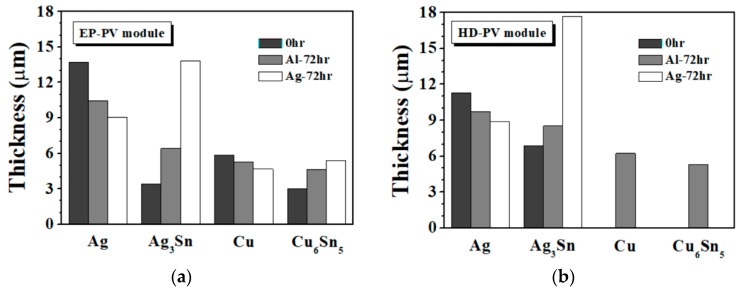
Variations in Ag, Ag_3_Sn, Cu and Cu_6_Sn_5_ thickness of (**a**) the EP and (**b**) the HD PV modules under various bias conditions.

According to the aforementioned results, the variations in the Ag electrode, Ag_3_Sn, Cu and Cu_6_Sn_5_ layer thicknesses of the EP and the HD PV modules with different bias conditions are summarized in Figure 8. Before biasing (0 h), the EP PV ribbon used a lower reflow temperature (265 °C), and thus decreased the growth of Ag_3_Sn IMC and the consumption of the Ag electrode. Ag-direction biased PV modules (EP and HD) had a higher growth behavior of Ag_3_Sn and Cu_6_Sn_5_ IMCs, which is attributed to the driving force direction of the electromigration. In particular, Ag_3_Sn IMC over-grew after Ag-direction biasing, increasing the series resistance of the module. Therefore, both of the PV modules in Al-direction biasing have better conductive efficiency (Table 3). Compared with the HD PV module, the Al-direction biased EP PV module had lowered the growth behavior of IMCs (Ag_3_Sn and Cu_6_Sn_5_), which is attributed to less Sn solder leading to a lower growth reaction of IMC. Thus, the increase in the series resistance of the EP PV module (26%) was lower than that of the HD PV one (34%). The series resistance of the hot-dipped Sn-50Zn PV module after biasing for 72 h was also measured (Table 3). The increase in series resistance of the Sn-50Zn PV module (14%) was lower than that of the EP PV one (26%), which is associated with the former not forming IMC at the solder/Al interface [16]. However, a high-Zn-content solder has a poor oxidation resistance in liquid state and high-cost issues [17]. The electroplating process can resolve the oxidation problem of Zn-containing solder, and reduce the cost of solder. Enhancing the conductivity of the Al ribbon and decreasing the over-growth behavior of IMC in the electroplated PV module can reduce the series resistance of the module structure, possessing a potential for solar cell applications.

## 4. Conclusions

1. This study uses an Al ribbon in place of a Cu ribbon, and fabricates the Cu∙Zn/Al ribbon via the electroless plating and electroplating processes, providing a connection between a solder and Al ribbon.
2. The signal of the Cu layer in the HD PV module did not appear because the Cu layer remelted into a liquid Sn solder during the hot-dip process. Under Al-direction biasing duration, Cu atoms continually accumulating and diffusing for more than 24 h would form the Cu layer and the Cu_6_Sn_5_ layer.
3. The Al-direction biased EP PV module had the same driving force (J_Cu (TM)_, J_Sn (EM)_) direction, which decreased the growth behavior of IMCs (Ag_3_Sn, Cu_6_Sn_5_). Under Ag-direction biasing condition, both the EP and the HD PV modules had higher series resistances, due to the electromigration effect leading the rapid growth of Ag_3_Sn and Cu_6_Sn_5_ IMCs.
4. Using an electroplated PV Al-ribbon can reduce the amounts of required solder, Ag paste and the cost of metal ribbon. Less solder reduced the growth reaction of IMCs, and had a better conductive efficiency of the PV module.

## Figures and Tables

**Table 1:** Chemical composition of the 1050 Al-ribbon.

Element	Al	Si	Fe	Zn	Ti	V
Wt.%	Bal.	0.09	0.21	0.01	0.02	0.02

**Table 2:** Series resistance of metal Al, Cu∙Zn/Al, Sn/Cu∙Zn/Al (EP) and Sn/Cu∙Zn/Al (HD) ribbons.

Specimen	Resistance (Ω)
**Al**	6.36 × 10^−2^
**Cu∙Zn/Al**	6.37 × 10^−2^
**Sn/Cu∙Zn/Al (EP)**	6.69 × 10^−2^
**Sn/Cu∙Zn/Al (HD)**	7.06 × 10^−2^

**Table 3:** Series resistances of Al-ribbon with different coated solders as a function of biased durations.

PV Modules	Current Direction	Resistance (Ω)		Increase (%)
		0 h	72 h	
**Electroplated Sn**	Al	6.69 × 10^−2^	8.43 × 10^−2^	26
	Ag		1.0 × 10^−1^	49
**Hot-dipped Sn**	Al	7.06 × 10^−2^	9.47 × 10^−2^	34
	Ag		1.01 × 10^−1^	43
**Sn-50Zn**	Al	3.6 × 10^−2^	4.2 × 10^−2^	14

## References

[1] Chen K.J., Hung F.Y., Lui T.S., Chen L.H., Qiu D.W., Chou T.L. (2014). Microstructure and electrical mechanism of Sn-xAg-Cu PV-ribbon for solar cells. Microelectron. Eng..

[2] Chen K.J., Hung F.Y., Lui T.S., Chen L.H., Chen Y.W. (2015). A study of green Sn–xZn photovoltaic ribbons for solar cell application. Sol. Eng. Mater. Sol. Cell..

[3] Abd El-Rehim A.F., Zahran H.Y. (2017). Investigation of microstructure and mechanical properties of Sn-xCu solder alloys. J. Alloy. Comp..

[4] Bae K.S., Kim S.J. (2002). Microstructure and adhesion properties of Sn-0.7Cu/Cu solder joints. J. Mater. Res..

[5] Li Z.Q., Belyakov S.A., Xian J.W., Gourlay C.M. (2018). The Influence of Primary Cu_6_Sn_5_ Size on the Shear Impact Properties of Sn-Cu/Cu BGA Joints. J. Electron. Mater..

[6] Yoon J.W., Jung S.B. (2005). Interfacial reactions between Sn–0.4 Cu solder and Cu substrate with or without ENIG plating layer during reflow reaction. J. Alloy. Comp..

[7] Chen Z., Tian W., Li J., Zhu W. (2018). Intermetallic Growth Induced Large-Scale Void Growth and Cracking Failure in Line-Type Cu/Solder/Cu Joints Under Current Stressing. J. Electron. Mater..

[8] Zeng G., McDonald S., Nogita K. (2012). Development of high-temperature solders: Review. Microelectron. Reliab..

[9] Lan G.A., Lui T.S., Chen L.H. (2011). The role of eutectic phase and acicular primary crystallized Zn phase on electrification-fusion induced fracture of Sn-*x*Zn solder alloys. Mater. Trans..

[10] Lin K.L., Wen H.L., Liu T.P. (1998). The microstructures of the Sn-Zn-Al solder alloys. J. Electron. Mater..

[11] Pstruś J., Fima P., Gancarz T. (2012). Wetting of Cu and Al by Sn-Zn and Zn-Al eutectic alloys. J. Mater. Eng. Perform..

[12] Yu C.H., Lin K.L. (2005). Early stage soldering reaction and interfacial microstructure formed between molten Sn-Zn-Ag solder and Cu substrate. J. Mater. Res..

[13] Haddadi F., Strong D., Prangnell P.B. (2012). Effect of zinc coating on joint properties and interfacial reactions in aluminum to steel ultrasonic spot welding. JOM.

[14] Palenskis V. (2013). Drift mobility, diffusion coefficient of randomly moving charge carriers in metals and other materials with degenerated electron gas. World J. Condensed Matter Phys..

[15] Shewmon P. (2016). Thermo- and Electro- Transport in Solids. Diffusion in Solids.

[16] Lin C.W., Hung F.Y., Lui T.S., Huang D.J. (2018). Study on characteristics of interfacial microstructure and electrical current mechanism in Sn-*x*Zn/Al photovoltaic modules. Sol. Energy.

[17] Jiang J., Lee J.E., Kim K.S., Suganuma K. (2008). Oxidation behavior of Sn-Zn solders under high-temperature and high-humidity conditions. J. Alloy. Comp..

